# Epidemiological, genetic and epigenetic aspects of the research on healthy ageing and longevity

**DOI:** 10.1186/1742-4933-9-6

**Published:** 2012-04-23

**Authors:** Alberto Montesanto, Serena Dato, Dina Bellizzi, Giuseppina Rose, Giuseppe Passarino

**Affiliations:** 1 Department of Cell Biology, University of Calabria, Ponte Pietro Bucci cubo 4 C, 87036 Rende, CS, Italy

**Keywords:** Ageing, Longevity, Genetic variation, Epigenetic modifications

## Abstract

Healthy ageing and longevity in humans result from a number of factors, including genetic background, favorable environmental and social factors and chance.

In this article we aimed to overview the research on the biological basis of human healthy ageing and longevity, discussing the role of epidemiological, genetic and epigenetic factors in the variation of quality of ageing and lifespan, including the most promising candidate genes investigated so far. Moreover, we reported the methodologies applied for their identification, discussing advantages and disadvantages of the different approaches and possible solutions that can be taken to overcome them. Finally, we illustrated the recent approaches to define healthy ageing and underlined the role that the emerging field of epigenetics is gaining in the search for the determinants of healthy ageing and longevity.

## Background

The past few decades witnessed a growing social and scientific interest in studies on human ageing and longevity. This interest is primarily due to the social burden connected to the extraordinary increase of the elder population in developed countries, which implies an increase of the subjects which are not autonomous and are affected by invalidating pathologies [1,2]. In Italy, for instance, in 1961 the population aged 65 and older was 4.8 million (9.5% of the total population), while in 1981 this number increased up to 7.5 million (13.2% of the total population) and in 2011 it grew up to 12.3 million (20.3% of the total population). In addition, the population aged 90 and older is growing at a faster pace as it has triplicated in the last 20 years (data from population Census and from http://www.istat.it). Proportionally, life expectancy at birth increased from a medium value of 44 years (44.2 for males and 43.7 for females) in 1905 to more than 80 years (79.4 for males and 84.5 for females) in 2011. Similar figures are reported for all developed countries, while in developing countries life expectancy grows very fast as soon as infant mortality is reduced, with the exception of some areas, namely in Africa, where AIDS infection dramatically affects life expectancy of adults [2].

Epidemiological evidence for a genetic component to variation in human lifespan comes from twin studies and family studies. By comparing life span in twins, researchers have found that approximately 25% of the overall variation in human lifespan can be attributed to genetic factors [3-5], which become more relevant for extreme longevity [6]. Conditioning factors, that arise in the first part of life (socio-economic state of parents, education and month of birth, which has been found to reflect the environmental conditions during the prenatal and early postnatal period), account for another 25% of such variability; life circumstances at adult and old age (including socio-economic status and medical assistance) may account for about the remaining 50% [7].

Family-based studies demonstrated that parents, siblings and offspring of long-lived subjects have a significant survival advantage when compared with the general population [8-12]. Moreover, these studies indicated that long-lived individuals and their children experienced a lower incidence of age related diseases and a higher degree of physical functioning and autonomy, when compared to appropriate selected controls [13-15]. However, how much of this reported survival advantage is due to common genetic factors or to a shared environment remained unclear. By using the original approach to adopt an intra-family control group, two different studies [16,17] confirmed that a substantial contribution in the familiarity observed in the above cited works was attributable to genetic variation, so prompting the research to deeply investigate the genetic variants favoring human longevity.

In this paper we will review the literature on the studies on the genetic of human longevity and the discussions there has been on the different approaches that can be used in this field. In addition, we will report the new approaches that have been proposed to define the healthy ageing, as the correct definition of healthy ageing is the first step to understand its genetic basis. Finally, we will outline some recent advance in the epigenetic studies of ageing, as epigenetics, a bridge between genetics and environment, might explain many aspects of ageing and longevity.

### Genetic variability and human longevity

The studies aimed to understand the genetic basis of longevity in humans have been carried out under the hypothesis that unfavorable genotypes should be dropped out of the population by a sort of "demographic selection" [18] which finally results in an enrichment of favorable genotypes in the gene pool of long lived people [19-21]. These studies have preliminarily faced the difficulty of clearly defining the phenotype under study. In fact, longevity is a dynamic phenomenon, where the definition changes in relation to the individual birth cohort. Indeed, survival curves change with time, in relation to the birth year of the cohort, thus medium age at death progressively increases with time modifying the number of subjects who can be defined as "long lived" [7,22]. In this frame, demographic analyses allowed to show that around the age of 90 years there is a clear deceleration of the age related mortality rate [23], suggesting that the subjects surviving to this age might be considered the long lived subjects who have survived the "demographic selection" mentioned above [24].

To date, many approaches have been adopted in order to disentangle the genetic from the environmental effects on human longevity, ranging from different sample design to data analysis approaches [25]. Among the different sampling strategies adopted in the field of human longevity research, a first distinction should be made between family-based and population-based studies.

#### Family-based studies

At family level, the ASP design represents the typical nonparametric strategy allowing both linkage and association to be tested [26]. At population level, cross-sectional (or case-control) cohort (longitudinal or follow-up) and case-only studies represent the most common design strategies providing important insights into the genetics of human longevity. Family-based designs show the unique advantages over population-based designs, as they are robust against population admixture and stratification. On the other hand, it is evident the difficulty to collect enough families, especially for late-onset complex traits such as lifespan, in which parental genotype information is usually missing. Despite these problems, non-parametric linkage analysis was attempted to localize genes implicated in human longevity. One of the first attempts to identify genetic regions co-segregating with the longevity phenotype by using an ASP approach has been carried out by Puca and co-workers [27]. Scanning the whole genome by applying non-parametric linkage analysis to long-lived sibpairs from USA they reported a region on chromosome 4 that could possibly harbor a gene affecting human longevity. In a following association-based fine-mapping experiment of the region, *MTTP *was identified as the gene most probably responsible for the observed linkage peak reported [28]. However, the association observed in this sample could not be replicated neither in a larger French sample of long-lived individuals nor in a sample of German nonagenarians and centenarians [28,29].

Among the studies using an ASP approach it is worth noticing the original study design adopted in the ECHA project [30]. The authors, by using cousin-pairs born from siblings who were concordant or discordant for the longevity trait, analyzed two chromosomal regions already known to encompass longevity-related genes. Although no significant differences emerged between the two groups of cousin-pairs (probably due to insufficient sample size) this study provided important insights to better dimension future sampling campaigns to study-genetic basis of human longevity. In particular the GEHA project [31] was launched in 2004 and was aimed to the sampling of an unprecedented number (2500) of nonagenarians sib-pairs from all over the Europe, to be analyzed for selected chromosomal regions previous related to the longevity trait, and for discovering new regions by a whole genome approach. Behind the scientific results still to be published, GEHA clearly represents an example of standard recruitment methodology, both for collecting biological samples and phenotypic information by home-based questionnaires, the last very crucial for the definition of phenotype [31].

#### Population case-control studies

Population case-control studies comparing long lived samples with younger controls of the same population may provide a powerful and more efficient alternative, especially when associated to the recent advances in genomic and statistical techniques. They are more powerful than family designs for detecting genes with low effect and gene-gene interactions [32]. However, these cross-sectional studies may suffer from the lack of appropriate control groups, as cohort specific effects may confound comparisons between very old people (for example centenarians) and younger cohorts [33]. The problem is hindered by the rapid changes of human societies that increase the level of population heterogeneity, thus introducing a further complicating factor. To cope with these problems, algorithms which integrate genetic and demographic data have been proposed [22,24,34,35]. Genetic-demographic methods allow the estimation of hazard rates and survival functions in relation to candidate alleles and genotypes. In such a way it is possible to compare survival functions between individuals carrying or not carrying a candidate allele or genotype without introducing arbitrary age classes, and taking into account cohort effects in mortality changes. Furthermore, the addition of demographic to genetic data not only is able to reveal sex- and age-specific allelic effects, but also permit a rational definition of the age classes to be screened [24]. Moreover, from the application of genetic-demographic model to longevity association studies, it emerged that genetic factors influence human survival in a sex- and age-specific way. In fact, in agreement with demographic data, genetic variability plays a stronger role in males than in females and in both genders its impact is especially important at very old ages [6,17,24].

#### Multi-locus approaches

Most gene-longevity association studies have focused on a single or a few candidate genes. However, common genetic variants with important effects on human longevity are unlikely to exist because of the rather low genetic contribution to the trait. In addition, given the complexity of the trait, the main effects of the individual loci may be small or absent, while multiple genes with a small effect may interact in an additive manner and affect survival at old ages. In such a case, a single-locus approach may not be suitable, failing in finding positive results of associations. Thus, given the technical improvement of typing techniques, multi-locus association approaches which takes into account epistatic interactions among different genes, have become of age [36].

These approaches represent specific and important statistical challenges. A flexible framework to tackle these challenges and for modeling the relationship between multiple risk loci and a complex trait makes use of logistic regression techniques [24,37]. Since from a statistical point of view epistasis corresponds to an interaction between genotypes at two or more loci, the same regression techniques have been easily extended to the analysis of gene-gene and gene-environment interactions in complex phenotypes, both at genome-wide and smaller scale studies level [38,39].

In some studies, different loci clustered in haplotypes are analyzed. In general haplotype-based association analysis brings new possibilities and difficulties. They exhibit more power than single-marker analysis for genetic association studies since they incorporate linkage disequilibrium information [40-42]. Conversely, the main difficulty is that haplotypes are often not directly observable, especially for late-onset complex traits such as lifespan, owing to phase uncertainty. Methods based upon likelihood can be extended to deal with kind of problem, most conveniently by use of the EM algorithm. Among these, the score tests proposed by Schaid et al. [43] are the most popular. Among the methods developed for haplotype-based multi-locus analysis of human survival, the original studies carried out by Tan et al. involving both cross-sectional [44] and cohort [45] designs studies of unrelated individuals are worth noticing.

Further improvements in high-throughput technology, associated to the recent advances in genomic knowledge, have made whole genome genotyping (> 100,000 SNPs) more accessible. Indeed, GWAS are at present widely used to find genetic variants contributing to variation in human lifespan [27,46-52]. In particular, Sebastiani and co-workers, consistently with the hypothesis that the genetic contribution is largest at the oldest ages and that long-lived individuals are endowed with multiple genetic variants with a single small effect, undertook a genome-wide association study of exceptional longevity, building a genetic profile including 281 SNPs able to discriminate between 800 centenarians cases and 900 healthy controls. The "genetic signatures of exceptional longevity" and relative subject-specific genetic risk profile which were obtained can provide important insights to dissect the unique complex phenotype into sub-phenotypes of exceptional longevity.

From a statistical point of view, the analysis of GWAS data presents several statistical challenges including data reduction, interaction of variables and multiple testing. Although these challenges are new to statistics, the magnitude of the present datasets is unprecedented.

After all these considerations, the most reasonable approach, for taking into account a great number of single polymorphisms spread along the genome without losing the biological relevance of candidate genes in biochemical pathways, which may be reasonably related to the trait, seems to be to use a candidate regions approach combined with a minimal number of "tagging" SNPs, efficiently capturing all the common genetic variation in the assayed genomic region [24,53-56]. This hybrid tagging-functional approach, by selecting the maximally informative set of tag SNPs in candidate-gene/candidate region for an association study, promises to shed a light in the genetic determinants of complex traits in general, and hopefully in human longevity too [57].

#### Candidate genes and candidate pathways in human longevity

By using the approaches described above, many candidate genes have been investigated to identify alleles that are either positively or negatively selected in the centenarian population as consequence of a demographic pressure. For many years, genetic analyses were focused on single genetic variants, by using the classical "candidate gene" approach. Candidates were found among human orthologous of experimental model genes, where the existence of specific mutations (*age-1, daf2, sir2, methuselah, p66*) able to extend or reduce lifespan has been reported [58-62]. In laboratory models, all the longevity genes identified have primary roles in physiological processes and especially in signal transduction; therefore it seems that natural selection does not select for genes that cause ageing in these organisms, but rather ageing occurs as a result of pleiotropic effects of genes that specify other fundamental processes.

In providing these insights, invertebrate studies motivated a lot the search for human genes involved in longevity and provided candidate genes, sometime successfully found associated with human longevity too (i.e. *KLOTHO, FOXO3a, SIRT3*; *UCPs; *[20,63-66]. However, these studies revealed also many challenges and claimed for caution that should be used when investigating human candidate genes identified by their orthology in animal models [33]. Another important category of candidate genes for ageing research are those involved in age-related diseases (in particular, cardiovascular diseases, Alzheimer's, cancer and auto-immune diseases) and genes involved in genome maintenance and repair (in particular, those involved in premature ageing syndromes such as Werner syndrome). The underlying hypothesis is that long lived persons should not present in their DNA any risk factors involved in pathologies. On the contrary, long lived individuals harbor genetic risk factors for age-related diseases [67,68] as recently underlined also by GWAS data, reporting as very long lived individuals share the same number of risk alleles for coronary artery disease, cancer, and type 2 diabetes than younger controls from the same population, thus suggesting that human longevity is not compromised by the cumulative effect of a set of risk alleles for common disease [69]. These studies support the existence of buffering mechanisms operating in the determination of human longevity, probably through the presence of favorable genotypes contrasting the deleterious effect of age-related disease genes: as a result, the frequency of deleterious genotypes may increase among individuals with extreme lifespan because their protective genotype allows disease-related genes to accumulate [70].

Recently, from the study of a single gene and starting again from the evidences in experimental models, which suggest the existence of evolutionary conserved networks that regulates lifespan and affects longevity across species, research moved into the study of whole metabolic pathways, where to find candidate genes for human longevity. From worms (*C. elegans*), to fruit flies (*Drosophila*), and mammals (*mouse*), pathways related to the regulation of energy homeostasis, cell maintenance, nutritional sensing, stress response signalling to internal or external environmental insults, by an efficient non inflammatory response, and DNA repair/maintenance have been shown to critically modulate lifespan [62,71] so harboring interesting candidate genes for longevity research. The insulin/IGF-1 pathway and downstream effectors, such as FOXO, are among of the most promising in this sense. Mutations affecting this pathway show effects on longevity from invertebrates to mammals, with several longevity mutants altering key components of the pathway, as for example the increased lifespan of mice heterozygous for the IGF1 receptor knockout1 [72]. Moreover, the downstream transcription factor DAF-16 (FOXO) regulates the expression of several genes involved in stress resistance, innate immunity, metabolic processes and toxin degradation [73]. Other interesting pathways for human longevity are represented by the TOR signalling, a major nutrient-sensing pathway, whose genetic down-regulation can improve health and extend lifespan in evolutionarily distant organisms such as yeast and mammals [74] and the recently deep investigated UCP pathway, a family of inner mitochondrial membrane proteins responsible for uncoupling substrate oxidation from ATP synthesis, whose expression was demonstrated to affect lifespan from fruit flies to mouse, somehow mimicking the metabolic and lifespan effects of caloric restriction (see [65] and references therein).

In humans, the most relevant results found by association studies in long lived cohorts, identified genes involved in GH/IGF-1/Insulin signaling (*GHR, IGF1R, FOXO3A*), antioxidant (*SOD1, SOD2, PON1, FOXO3A*), inflammatory (*IL6, CETP, Klotho*) pathways, silencing (*SIRT1 and SIRT3*), elements of lipid metabolism (*APOE, APOB, ACE, APOC3, MTTP*) and stress resistance (*HSPA1A and HSPA1L) *[[19,33,75-81] and references therein]. However, most of these results, with the exception of *APOE *and *FOXO3A*, were not reproduced in some of the replication studies [29,82], probably because of problems in study design and publication bias. This points to the need of larger populations for case-control studies in extreme longevity, use of replication cohorts from different populations and appropriate multiple comparisons tests to reduce the bias of these kind of studies [83].

#### Functional consequences of genetic variants associated with human longevity

Coupled with the rapid advances in high-throughput sequencing technologies, it is now feasible to comprehensively analyze all possible sequence variants in candidate genes segregating with a longevity phenotype and to investigate the functional consequences of the associated variants. A better understanding of the functional genes that affect healthy longevity in humans may lead to a rational basis for intervention strategies that can delay or prevent age-related diseases. Genome-wide expression profiles in different tissues reported changes of gene expression occurring with age. In this sense, two main works deserve attention. Kerber and collaborators, who analyzed the gene expression profiles of 2151 house-keeping genes in cultured cell lines from 104 adults belonging to 31 Utah families, aged 57-97 years, searching for stable variation in gene expressions that affect or mark longevity. They found different genes exhibiting associations with either mortality or survival [84], 10% decreased in expression with age, and 6% increased with age. Significant association both with age and survival was observed for CDC42, belonging to the DNA repair pathway and CORO1A, an actin-binding protein with potentially important functions in both T-cell mediated immunity and mitochondrial apoptosis [85], underlying the potential importance of these metabolic pathway in longevity determination. More recently, Slagboom and co-workers [81] compared the expression profiles of candidate genes in a limited number of subjects (50 for each group) among offspring of long-lived subjects and their partners. Among the differentially expressed genes, they observed a decreased expression of genes in the mTOR pathway in the members of long-lived families. Although it is likely that epigenetic factors may also play a large role [86] and the results should be replicate in a larger sample, it is clear that by combining the molecular epidemiological studies with a genomic approach may provide a step further towards the identification of early and possibly causal contributions to the ageing and human longevity process.

#### The special case of the mitochondrial genome

Human ageing is characterized by a gradual reduction in the ability to coordinate cellular energy expenditure and storage (crucial to maintain energy homeostasis), and by a gradual decrease in the in the ability to mount a successful stress response [87]. These physiological changes are typically associated with changes in body composition (i.e. increase in fat mass and the decline in fat-free mass), and with a chronic state of oxidative stress with important consequences on health status [88]. Mitochondrial function is crucial in these processes, being mitochondria the main cellular sites controlling energy metabolism and the redox state. Mitochondria are considered as key components of the ageing process, playing a pivotal role in cell survival and death since they contribute to many cellular functions, including bioenergetics, protection from oxidative damage, maintenance of mtDNA and cell death [89]. Moreover, in addition to ATP production, mitochondria form a complex metabolic network which is crucially involved in glucose sensing/insulin regulation, intracellular Ca2+ homeostasis and many metabolic signaling pathways [90]. On the other hand, mitochondria are the major producers of ROS and at the same time targets of ROS toxicity. Consequently, the maintenance of a healthy mitochondria population represents a major target of a well functioning organism, for preserving many physiological functions, such as neurotransmission [91]. Starting from the important role of this organelle in the cell homeostasis, the effect of both inherited and somatic variability of mtDNA in ageing and longevity has been deeply investigated, resulting complex and sometime controversial [92].

An accumulation of mtDNA somatic mutations occurs with age, and many studies have reported an association between mtDNA mutations and ageing, particularly in post-mitotic neuronal cells [93]. A number of mutations not associated to diseases have been fixed along time in the mtDNA sequence, to form a series of population-specific lineages that can be identified by the presence of conserved groups of haplotypes (haplogroups). These germline inherited mtDNA variants (haplogroups and their subclassification into subhaplogroups on the basis of specific mutations identified by sequence analysis of the D-loop region) are used for tracing back the origin of populations or in forensic analyses [94]. Considered biochemically neutral, the mtDNA inherited variability is probably able to differently modulate the mitochondrial metabolism [95]. mtDNA haplogroups have been positively associated with mitochondrial, complex diseases and ageing [96,97]. In particular, in Caucasians the haplogroup J is over-represented in long-living people and centenarians, thus suggesting a role for this mtDNA variant in longevity [98]. As for somatic variations, tissue specific mutations occurring in the mtDNA control region have been proposed to provide a survival advantage, i.e. the C150T transition [99]. Data analyzing the occurrence and accumulation of C150T mutation in centenarians' relatives and long lived sib pairs demonstrated a genetic control on the mtDNA heteroplasmy (i.e. the presence of different molecule of mutant/wild type mtDNA), suggesting the existence of nuclear genetic factor influencing their accumulation [100,101]. The observation that the nuclear genome contributes to mtDNA heteroplasmy marks the importance of the mitochondrial-nucleus cross-talk in modulating mitochondrial function and cellular homeostasis and, consequently, quality of ageing and lifespan [102]. Such a nuclear-mitochondrial cross-talk was firstly observed in yeast, where a compensatory mechanism, named "retrograde response" has been described, allowing to mutant strains of yeast to cope with mtDNA impairments by up-regulating the expression of stress-responder nuclear genes [103] and leading to a significant increased lifespan.

The first experimental evidence that a similar mechanism has been maintained in higher organisms, including humans, comes from cytoplasmic hybrid or cybrid experiments (i.e. cell lines differ only in the source of their mtDNA), where it was found that cells characterized by different mtDNA haplogroups, differently expressed stress responder nuclear genes [104,105], thus suggesting that the retrograde response mechanism may represent an evolutionary conserved strategy for the age-related remodeling of organismal functions.

On the whole, although the involvement of mtDNA variability in ageing and longevity is undisputed, the role of mtDNA and its mutations, either inherited or somatically acquired, in human longevity is far from being clear. The use of high-throughput technologies and the extensive analysis, possibly at the single cell level, of different tissues and cell types derived from the same individual will help in disentangling the complexity of mtDNA in ageing and longevity.

#### The maintenance of telomere length

Genomic instability has been widely recognized as a crucial mechanism in both ageing and age-related diseases. The progressive shortening of telomeres, probably the most important marker of chromosome integrity, is associated with increased risk of several age-related disease comprised cancer and mortality [106,107]. Telomeres play a central role in maintaining the chromosome stability, preventing the inappropriate activation of DNA damage pathways, and regulating cell viability, by triggering signals of ageing to normal cells to senesce when telomeres stop their functioning [108]. Their length is controlled by telomerase. In normal human cells telomerase is expressed in stem cells, cells that need to actively divide (like immune cells) and is barely, or not expressed at all in differentiated somatic cells. However, higher expression of telomerase strongly correlated with carcinogenesis, with approximately 85%-90% of human cancers showing higher enzymatic activity [109]. Furthermore, suppression of telomerase activity in telomerase-positive cancer cells results in cell death and tumor growth inhibition [110], highlighting the critical role of telomerase in facilitating and enabling cancer cell proliferation. On the contrary, high telomere stability correlates with human longevity, with healthy individuals showing significantly longer telomeres than their unhealthier counterparts [68,111]. Longer telomeres are associated with protection from age-related diseases, better cognitive function and lipid profiles, thus may confer exceptional longevity [112]. The understanding of the complex tradeoff between cancer development and long life in relation to telomere maintenance represents one of the most intriguing challenges for researchers in human longevity. Considering these evidences, centenarians may represent the best example of a well preserved telomere length, harboring the right compromise of having longer telomeres and never have been affected by cancer or survived to a cancer episode, so may represent optimal control population for association studies aimed to disentangle the complex role of telomere maintenance in age-related diseases and ageing.

### Successful ageing and frailty

Although ageing is a general phenomenon, it is clear that a great inter-individual variability on the rate and quality of ageing can be observed [33]. Following the paradigm "Centenarians as a model for healthy ageing", centenarian studies have allowed to identify a number of characteristics associated with extreme longevity. For example, nonagenarian and centenarian men are generally taller and heavier than women of corresponding age and have a greater amount of muscle and trunk fat, whereas women are small and show a marked peripheral adipose distribution [113]. Furthermore, food preferences, marital status, personality and coping strategies, levels of family support, and education have all been linked with successful late-life ageing [113-118]. However, whether centenarians represent healthy ageing still remains an open question. Franceschi and co-workers recognized that on the basis of their functional status centenarians might be classified into three categories [119]. Most of them suffer of disabilities or diseases [120], and in general they experience a loss of independence [1], but a minority of them are still in quite a good health. According to this perspective, centenarians are not the most robust subjects of their age cohort, but rather those who better adapted and re-adapted from both biological and non-biological point of view, and in general they constitute a very heterogeneous group of individuals [119]. Hence several studies searched for indicators of health and functional status in old and very old subjects by which objective phenotypes could be defined [121-126]. From these studies the concept of frailty emerged as a distinct clinical entity, characterized by a state of vulnerability for adverse health outcomes, such as hospitalization or death, and therefore correlated to co-morbidity, disability and increased mortality hazard [127]. The "frailty" syndrome of the elderly is mainly correlated to the decline of homeostatic capacity of the organism, which implies the decline of different physiological systems, such as the neuromuscular and the cognitive systems, and which leads to a significant increase of disability, comorbidity and death risk [121]. The frailty declines with age and make less efficient the metabolic pathways for the conservation, the mobilization and the use of the nutrients, thus representing the physiologic precursor and etiologic factor in disability, due to its central features of weakness, decreased endurance, and slowed performance [121]. Therefore, the identification of a precise frailty phenotype could help to recognize homogeneous population groups enriched of genetic risk factors predisposing to a poor quality of ageing. How to measure frailty? First of all, because population specificity was demonstrated in the quality of ageing [128], it is necessary to carry out population specific surveys to define the tools which are able to highlight within each population groups of subjects with homogeneous "ageing phenotype". Among the methodologies used to classify homogenous subgroups within each population, the cluster analysis proved to be very useful to identify groups of subjects homogeneous with respect to chosen variables. As for the parameters to be used for the classification, cognitive, psychological and functional measures turned out to be the most effective to identify the frailty phenotype, since these parameters condense most of the frailty cycle that occurs in the elderly [122]. In particular, classification variables useful for grouping individuals respect their frailty status are represented by SHRS, ADL, HG strength and MMSE [129,130]. This kind of classification, which allows to define three main frailty groups (i.e. frail, pre-frail and non frail subjects), was firstly applied to a Southern Italian population, and proved to be able to foresee health status by the analysis of perspective survival. In particular, a longitudinal study showed a differential incidence of mortality after 18 and 36 months follow-up of the different groups identified [129]. The proposed classification was replicated in two large longitudinal Danish samples [130], where different ageing conditions had been previously described [128], confirming the predictive soundness after a 10-years follow up. In addition, in the same work the differential effect of distinct parameters on survival was estimated, founding that high values of HG and MMSE induced a higher probability of surviving, while being male, having a low ADL or a poor SRHS tended to reduce expected survival time. Furthermore, the presence of a genetic influence on frailty variance was suggested by the estimation of heritability of the frailty status, where it was found that the additive genetic component accounts for 43% of the overall variability of frailty levels between couple of twins. In line with previous findings, the estimate was higher in males than in females, consistent with the hypothesis that frailty status of men is more related to the genetic background while the frailty conditions of females are more dependent on environmental factors. In addition, as for lifespan, the influence of the genetic component on frailty status was found higher at advanced ages.

On the whole, this approach, which is based on population-specific data under study and does not use any a priori thresholds, may be very promising for an objective identification of frail subject. This may be a very important task for future societies, helping to address specific medical care, by tailoring treatments on the basis of the real needs of each single patient, especially of pre-frail and frail older patients with multiple chronic conditions and reduced life expectancy, finally preventing the effects of frailty.

### The role of epigenetics in human ageing and longevity

Epigenetic modifications indicate the sum of heritable changes, such as DNA methylation, histone modification and miRNA expression, that affect gene expression without changing the DNA sequence [131]. It is becoming clear that epigenetic information is only partially stable and destined to change across the lifespan representing a drawbridge between genetics and environment. Epigenetic variations have been suggested to have an important role in cellular senescence, tumorigenesis and in several diseases including type-2 diabetes, cardiovascular and autoimmune diseases, obesity and Alzheimer disease [132]. A correlation between epigenetic DNA modifications and human lifespan has been shown by Fraga et al. [133], who found that global and locus-specific differences in DNA methylation in identical twins of different ages are influenced by environmental factors and lifestyle. Most studies demonstrated that ageing is associated with a relaxation of epigenetic control; from one side, a decrease in global cytosine methylation has been found during ageing both in vivo and in vitro studies, mostly due to the demethylation in transposable repetitive elements [134,135]. On the other hand, an age-related hypermethylation has been observed in promoter regions of specific genes, such as those genes involved in cell cycle regulation, tumor-cell invasion, apoptosis, metabolism, cell signaling and DNA repair, with a consequent decrease of correspondent mRNA levels, confirming the potential role of these pathways in human ageing [136-143]. Moreover, recent studies reported as different epigenetic profiles can be associated with a different quality of ageing. Bellizzi and co-workers [144], studying the distribution of methylation pattern in a sample of elderly subjects stratified according to their quality of ageing (described by their scores in specific functional, cognitive and psychological tests), found that the level of methylation is correlated with the health status in the elderly. In particular, a significant decrease in the global DNA methylation levels was associated with functional decline, suggesting that the relaxation of the epigenetic control in ageing is specifically associated with the functional decline rather than with the chronological age of individuals. These results confirm that epigenetic variations, which in turn depend on hereditary, environmental and stochastic factors, may play an important role in determining physiological changes associated to old age.

## Conclusions

Despite the enormous technical progresses, which allow to analyzed many single variants as well as the coordinated expression of many genes together by high-throughput platforms, many challenges still remain to be faced by the researchers trying to identify genetic and non genetic variants associated with human longevity. A close partnership between gerontologists, epidemiologists, and geneticists is needed to take full advantage of emerging genome information and technology and bring about a new age for biological ageing research. In addition, we believe that the next future will see much progresses in our understanding of the longevity trait, mainly coming from the integration of genetics and epigenetics information by multidisciplinary approaches, to the aim of obtaining an overall picture of what successful ageing is.

## Abbreviations

ACE: Angiotensin I converting enzyme; ADL: Activity of Daily Living; APOE/B: Apolipoprotein E/B; APOC3: Apolipoprotein C-III; ASP: Affected Sib-Pairs; ATP: Adenosine triphosphate; CDC42: Cell division cycle 42; CETP: Cholesteryl ester transfer protein; CORO1A: Coronin, actin binding protein, 1A; DNA: Deoxyribonucleic Acid; ECHA: European Challenge for Healthy Aging; EM: Maximum Estimation; FOXO3A: Forkhead box O3; GEHA: Genetics of Healthy Aging; GH: Growth Hormone; GHR: Growth hormone receptor; GWAS: Genome-Wide Association Studies; HG: Hand grip; HSPA1A: Heat shock 70 kDa protein 1A; HSPA1L: Heat shock 70 kDa protein 1-like; IGF-1: Insulin Growth Factor 1; IGF1R: Insulin-like growth factor 1 receptor; IL6: Interleukine 6; miRNA: MicroRNA; MMSE: Mini Mental State Examination; mRNA: Messenger RNA; mtDNA: Mitochondrial DNA; mTOR: Mitochondrial Target Of Rapamycin; MTTP: Microsomal Triglyceride Transfer Protein; PON1: Paraoxonase 1; ROS: Reactive oxygen species; SOD1: Superoxide dismutase 1, soluble; SHRS: Self-reported health status; SIRT1/3: SIR2-like protein 1/3; SNP: Single Nucleotide Polymorphism; SOD: Superoxide dismutase; TOR: Target Of Rapamycin; UCP: Uncoupling Protein.

## Competing interests

The authors declare that they have no competing interests.

## Authors' contributions

AM and SD wrote the first draft; subsequent drafts were written by DB, GR and GP who also supervised the review process; all the authors edited the manuscript and approved its final version.
